# Incidence of hypotension according to the discontinuation order of vasopressors: a matter of pharmacokinetics

**DOI:** 10.1186/s13054-018-2131-9

**Published:** 2019-03-12

**Authors:** Ross Freebairn, Brigitte Hollander

**Affiliations:** 1 Hawke’s Bay Hospital, Hastings, New Zealand; 2 Chinese University of Hong Kong, Hong Kong, China; 3 University of Otago, Dunedin, New Zealand

We read with interest the study by Jeon and colleagues on hypotension developing during withdrawal of the two vasopressor agents, norepinephrine or vasopressin, demonstrating a higher incidence of hypotension at the end of the first hour with changes in norepinephrine than vasopressin [1].

The half-life of norepinephrine is reported as being between 2 and 6.8 min [2]. One hour is more than eight half-lives, and thus the norepinephrine would be very, very close to a new steady state of infusion. Even if the 6.8 min half-life is used this makes greater than 99.7% of the change having occurred. As the drop in norepinephrine infusion rate is 33.3% of the total, the new serum level would be approximately 67% of the original value.

In contrast, the half-life of vasopressin is 30 min [3]. One hour following the change two half-lives would have past, the vasopressin level would be still dropping, and only three quarters of the total change would have occurred. As the vasopressin infusion rates are altered only by 10%, the new serum level of vasopressin would be 92.5% that of the original infusion.

Thus, the comparison is the effect of lowering one serum level of medications by 7.5% with lowering another serum level of medication by 33%, a four-fold difference. It could be concluded that the higher incidence in hypotension with norepinephrine may be due to its more dramatic relative drop in serum level than in the vasopressin group.

Pharmacokinetics principles would suggest that hypotension due to vasopressin infusion changes would take longer to be clinically manifested than those of norepinephrine. Not only does the study compare different proportional changes, it measures the effect in a time frame too short to see the full effect of vasopressin infusion changes.
